# Combining Mass Spectrometry and X-Ray Crystallography for Analyzing Native-Like Membrane Protein Lipid Complexes

**DOI:** 10.3389/fphys.2017.00892

**Published:** 2017-11-09

**Authors:** Felipe A. Montenegro, Jorge R. Cantero, Nelson P. Barrera

**Affiliations:** Laboratory of Nanophysiology and Structural Biology, Department of Physiology, Faculty of Biological Sciences, Pontificia Universidad Católica de Chile, Santiago, Chile

**Keywords:** mass spectrometry, native mass spectrometry, membrane protein, gas phase, ligand binding, molecular dynamics

## Abstract

Membrane proteins represent a challenging family of macromolecules, particularly related to the methodology aimed at characterizing their three-dimensional structure. This is mostly due to their amphipathic nature as well as requirements of ligand bindings to stabilize or control their function. Recently, Mass Spectrometry (MS) has become an important tool to identify the overall stoichiometry of native-like membrane proteins complexed to ligand bindings as well as to provide insights into the transport mechanism across the membrane, with complementary information coming from X-ray crystallography. This perspective article emphasizes MS findings coupled with X-ray crystallography in several membrane protein lipid complexes, in particular transporters, ion channels and molecular machines, with an overview of techniques that allows a more thorough structural interpretation of the results, which can help us to unravel hidden mysteries on the membrane protein function.

## Introduction

Membrane proteins (MPs) are not merely inserted in the lipid bilayer. Besides interacting with ligands (which includes lipids, nucleotides, ions, and drugs) to regulate their stability and function; they do not work as independent entities, instead, they interact with other proteins to become membrane complexes that require a defined lipid environment to carry out their cellular transduction pathways. Given their relevance to cellular physiology, MPs comprise around 60% of drug targets (Overington et al., 2006).

Stoichiometry of ligands in intact MPs is key to understand their role on the proper biological function; however structural methods such as X-ray crystallography (Moraes et al., 2014) and solid-state nuclear magnetic resonance (NMR) spectroscopy (Ding et al., 2013) encounter experimental difficulties because of protein solubility/heterogeneity and need high resolution to properly assign the nature of the small molecules. Additionally, X-ray crystallography provides location of small molecules bound to MPs but structural refinement for disordered ligands could produce ambiguity for the complete characterization of the binding site (Marsh and Páli, 2004). Furthermore, proteins are fast and dynamic molecules which could interfere with the necessary symmetry on protein crystals, delivering a snapshot in a particular condition that can be crystallized (Dill and MacCallum, 2012). Therefore, complementary structural methods can provide relevant insights into the nature of ligands bound to transmembrane (TM) regions. Especially, Mass Spectrometry (MS) has been widely used for small molecule identification in protein samples. However, only recently it has been applied to get simultaneously structural information on MPs and protein-ligand stoichiometry. Strikingly within only a few years MS on native-like MPs has characterized several transporters, ion channels, and molecular machines which contain specific small molecular species tightly bound to the TM domains (Barrera and Robinson, 2011; Bechara and Robinson, 2015).

In this perspective, we address current experimental methods, as well as the contribution of computational approaches, to study MPs and lipid-binding events, with a focus on recent MS findings in the field.

## Biophysical methods to analyze membrane protein-lipid binding

Apart from X-ray crystallography and NMR spectroscopy, there are other complementary methods that can characterize protein ligand interactions, such as dissociation binding constants (surface plasmon resonance, SPR), kinetics or thermodynamics of such interaction (isothermal titration calorimetry, ITC), and intra and intermolecular distances <10 nm (Forster resonance energy transfer, FRET). Nevertheless, their use on testing lipid species has been rather challenging due to the low solubility in physiological conditions, in particular once lipids are added exogenously after protein purification. Atomic force microscopy (AFM) can render a surface 3D image of MPs at physiological conditions, and even tracking their fast-conformational changes. Combined with Force Spectroscopy measurements, intramolecular or ligand-binding interactions can be quantified at single-molecule level (Shahin and Barrera, 2008; Suzuki et al., 2013). While many biophysical methods have been extensively reviewed (Vuignier et al., 2010; Fang, 2012; Pacholarz et al., 2012) and continuously developed for tackling the identification of ligand binding events in proteins, the vast majority of these reviews focus on drug discovery. Therefore, research focused on structural biology of cellular ligand-binding events in intact MPs has been just recently expanding.

**Figure 1:**
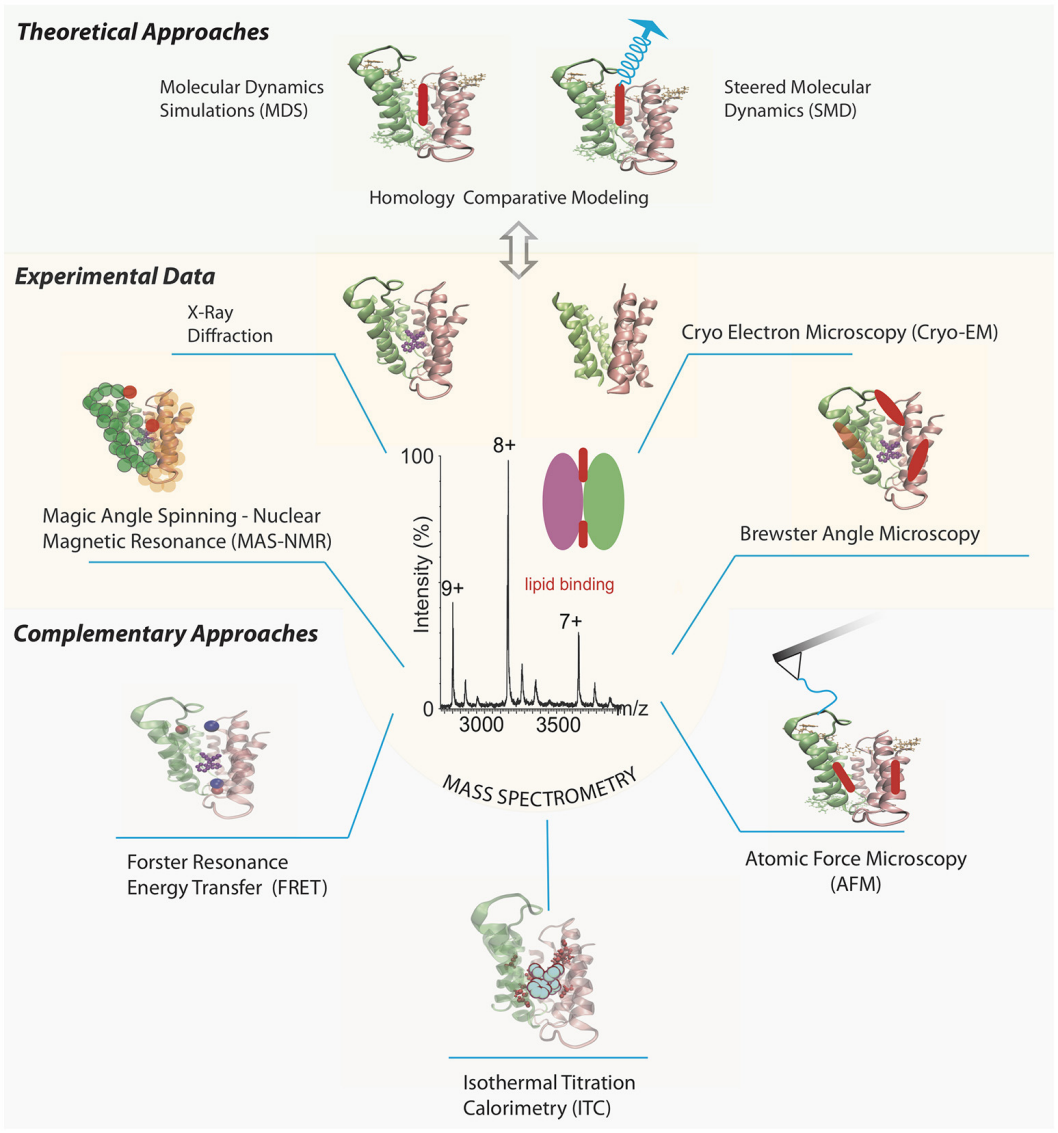
Selection of different biophysical methods aimed to determine structural and functional information of multidrug transporter EmrE bound to lipids. Different panels represent features of the chosen method, and EmrE transporter is drawn using the X-ray crystallized protein PDB ID 3B5D (Chen et al., 2007) in all cases, except for the cryo-EM structure where the PDB ID 2I68 was chosen (Fleishman et al., 2006). Mass spectra of EmrE is simulated based on previous results (Barrera et al., 2009) highlighting its relevance between experimental and theoretical methods to tackle the study of MP-lipid complexes. AFM tip chemically functionalized with a residue in one EmrE monomer to get stability properties of the MP-lipid interaction via force spectroscopy. Labelling pair of residues or one residue and a ligand to analyze overall protein structure via FRET and MAS-NMR experiments. SPR and ITC measurements to analyze the role of additional soluble ligands on the MP-lipid complex kinetics and thermodynamical properties, respectively. Brewster angle microscopy to characterize the stability between the MP and lipid interaction. MD and SMD (from a particular target atom in the lipid) simulations to obtain all-atom characterization of the MP-lipid complex, based on the collected experimental data. Lipids are drawn as red and blue colored ovoids. Tetraphenylphosphonium structure is colored in purple.

An interesting example of an MP analyzed by a series of structural experimental techniques is the small multidrug resistance transporter EmrE (Figure 1). Traditional methods such as cryo-electron microscopy (cryo-EM) and X-ray crystallography have resolved the structure of the EmrE dimer topology (Chen et al., 2007; Korkhov and Tate, 2008). Further analyses of the intact dimer have shown stoichiometric lipid binding and posttranslational modifications (PTMs) by MS (Barrera et al., 2009) and identification of a specific residue involved in the substrate binding site of tetraphenylphosphonium by magic angle spinning-NMR (Ong et al., 2013). The interaction between annular lipids and EmrE has also been tested using Brewster angle microscopy (Nathoo et al., 2013), which showed longer unsaturated chains of cardiolipin (CL) forming the most stable monolayer in the presence of EmrE. Once a MP-ligand complex has been identified, especially for those lipids already co-purified from a cellular expression system, complementary results for their effect over soluble ligand binding can be provided by AFM, SPR, ITC, and FRET. Altogether, these experimental techniques could benefit from theoretical studies including Molecular Dynamics simulations (MDS) and steered MD (SMD) for the lipid-membrane protein interactions (Kalinin et al., 2012; Shoura et al., 2014). It is becoming clear that a better in-depth understanding of the binding process requires a combination of different structural techniques, involving unambiguous identification of the ligand, its atomic location and dynamic mechanisms of the binding event.

## Mass spectrometry on lipid binding to native-like membrane proteins

MS has evolved from an analytical chemistry method to a structural biology technique. It requires an ionized sample in vacuum, going through electromagnetic fields, and being identified by their mass/charge ratio. MS uses two main technologies for ionization: MALDI (matrix adsorbed laser desorption ionization) or ESI (electrospray ionization). Of those, ESI -which relies on electric-induced droplet formation and desolvation- has been the most employed in structural studies of biomolecules since it can be easily coupled to liquid chromatography (LC-MS) and facilitate the structural integrity of the protein in vacuum (Konermann et al., 2014).

Back in 2001, Cohen and Chait reviewed the use of MS during different stages of protein crystallization as a help tool for identification of proteins (via peptide mapping) or domain cores; the detection of PTMs and examining degradation/oxidation of proteins after crystallization over long periods of time (Cohen and Chait, 2001). In those days, MS used inert gas to trigger the fragmentation of selected ion peptides (process denominated tandem MS, or MS/MS).

**Figure 2:**
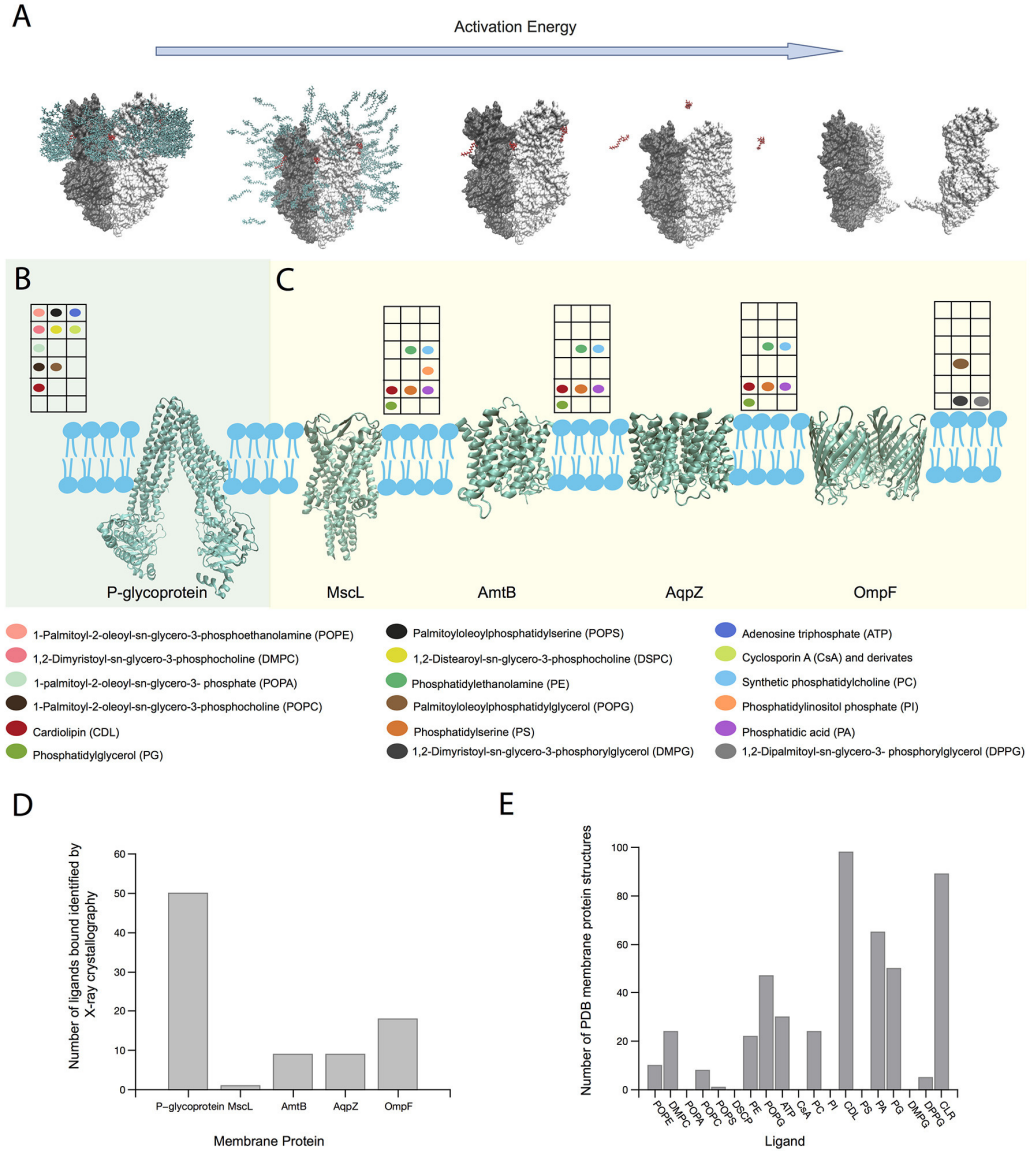
Role of MS on MP-lipid binding structural characterization. **(A)** Scheme of the release of an intact MP-ligand complex (MexB) from a micelle after increasing energy activation in the gas phase. Ligands and detergent molecules are colored red and green, respectively. **(B)** P-glycoprotein and its ligand interactions determined by native MS (Marcoux et al., 2013). **(C)** Variety of lipid interactions with MPs MscL, AmtB, and AqpZ (Laganowsky et al., 2014) and OmpF (Gault et al., 2016) determined by native MS. Color-coded circles show the presence of particular ligands studied in each MP by MS. The position of each circle in the grid matches with its position in the colored legend describing the ligand name. **(D)** Number of ligands bound to those MPs shown in **(B,C)**, which have been identified by X-ray crystallography until August 2017. **(E)** Number of crystallized MPs bound to ligands shown in **(B,C)**, including cholesterol (CLR), until August 2017. Note that ion bindings were not considered in the selection. Protein structures were generated using PDB IDs 2V50 (MexB) (Sennhauser et al., 2009), 4Q9H (P-gp) (Szewczyk et al., 2015); 2OAR (MscL) (Steinbacher et al., 2007); 1U7G (AmtB) (Khademi et al., 2004); 2O9F (AqpZ) (Savage and Stroud, 2007) and 3POQ (OmpF) (Efremov and Sazanov, 2012).

Since the last decade, MS has been extensively used for intact membrane macromolecular complexes to determine their stoichiometry, subunit arrangement, binding events and PTMs (Barrera et al., 2009; Wang et al., 2010; Zhou et al., 2011; Laganowsky et al., 2014; Gault et al., 2016). For MPs, several approaches to mimic the membrane environment have been used. The first approach to study native-like MP complexes was achieved using detergents in high concentrations (Barrera et al., 2008). The inert gas previously used for fragmentation, is now used to destroy the micelle, thus releasing an MP complex for native MS analysis, a process diagramed in Figure 2A. Considering that micelles only mimic the hydrophobic section of the membrane, other approaches to mimic a cell membrane were used in the following years (amphipols, bicelles, nanodiscs) (Marty et al., 2016). As small molecule binding such as lipids are needed to carry out an efficient protein function, only few methods have been used to identify the type and stoichiometry of these bindings. Experimental procedures in the purification and crystallization stages can sometimes delipidate the protein and therefore only lipids strongly bound to the TM domains survive to be identified by structural methods.

Fine-tuning of voltage and pressure settings of the mass spectrometer has allowed identification of lipids bound to the MPs, which were transferred from the cell membrane to the detergent micelle or nanodisc, thus preserving the MP-ligand interaction (Barrera et al., 2009; Barrera and Robinson, 2011; Zhou et al., 2011; Hopper et al., 2013; Laganowsky et al., 2013; Marcoux et al., 2013; Gault et al., 2016; Figure 2A). It is important to highlight though that the selection of the “membrane mimetic” strategy for the MP purification has practical issues in the MS analysis. While detergents cannot emulate lateral pressure profile or membrane curvature, that could reduce the MP stability, their removal from the complex requires lower collision energy. On the other hand, nanodiscs can maintain properties like membrane curvature or lateral pressure, but their removal requires much higher activation, preventing the identification of larger MP complexes (Hopper et al., 2013; Marty et al., 2016). So, there's a trade-off between simplifying the “membrane mimetic” environment to maintain native-like MP structure and the energy required to identify the MP.

The ionization process can be simulated through MD, assigning charge on basic residues (Friemann et al., 2009; Hall et al., 2012b) and calculating the solvent accessible surface area of the membrane complex (Morgner et al., 2012). Although it is clear that micelle protects the MP structure in vacuum (Barrera et al., 2008; Friemann et al., 2009; van der Spoel et al., 2011; Reading et al., 2015), the molecular mechanism of the protein release remains poorly understood. Specifically, it would be relevant to understand how lipids can still be bound to the protein after micelle destruction in the gas phase (Figure 2A). By steering MD simulations, Rouse et al. have shown that a MP-micelle complex in solution can be transferred to the gas phase, observing conformational changes in the micelle structure (Rouse et al., 2013). Hall et al performed MD simulations of SAP soluble protein in vacuum at increasing temperatures, from 300 to 800°K, in order to represent increasing activation energy in Ion Mobility-Mass Spectrometry (IM-MS) experiments, which resulted in a good correlation between the experimental and theoretical values (Hall et al., 2012a). This could imply that increasing temperature to trigger micelle destruction in MD simulations can be comparable to inert gas collisions, which should increase the micelle internal energy, making it unstable and finally dismantle it, releasing the protein in a native-like state.

## Mass spectrometry as a collaborative tool for crystallography

Considering lipid identification in native-like MPs, this high-resolution MS method shows important progresses leading to the understanding of structural features hidden on MPs. For example, on one hand, we can mention 3 MPs studied by MS prior to the knowledge of their atomic structure. MS has resolved 2 phosphatidylethanolamine (PE) molecules bound to the ATP binding cassette (ABC) transporter MacB dimer in 2009, probably located at the dimeric interface. In 2017, the MacB cryo-EM structure obtained at 3.3Å resolution did not show lipid binding perhaps due to the purification procedure and limited map local resolution (Fitzpatrick et al., 2017).

The resistance-nodulation division transporter MexB trimer was analyzed in 2009, where the presence of lipid clusters was identified using MS (Barrera et al., 2009). The same year a 3Å resolution crystal structure was available (Sennhauser et al., 2009), and an unidentified density was detected in the MexB pore domain, which was interpreted as maltose ring of a DDM detergent molecule, since no ligands were used in the crystallization process. Later, in 2013 a 2.7Å resolution crystal structure was published showing elongated electron densities in the central hole of the TM domain, which likely correspond to acyl chains of membrane phospholipids (Nakashima et al., 2013).

In 2015, the heterodimeric ABC exporter TmrAB was identified bound to negatively-charged phosphatidylglycerol (PG) and zwitterionic PE lipids, where PG showed a stronger affinity for the transporter. That same year, a cryo-EM structure of TmrAB was published with an 8Å resolution (Kim et al., 2015). A pocket in TmrB formed between two TM segments showed a density that the authors attributed to either detergent or lipid co-purified in the sample preparation steps. In 2017, a crystal structure with 2.7A resolution was available (Noll et al., 2017), which showed that pocket containing 5 arginine residues. MD simulations showed lipid interactions in the TmrB pocket, with the polar head interacting with the pocket while the aliphatic chains interacting with the hydrophobic part of the membrane, possibly showing a mechanism for lipid translocation, in accordance with the previous MS data.

In 2011, while studying the stochiometry of intact rotary adenosine triphosphatase (ATPase)/synthase from *Enterococcus hirae* via MS, 10 cardiolipins (CLs) were bound to the inside of the K10 ring in the enzyme. Interestingly these lipids were originally assigned to be 20 1,2-dipalmitoyl-phosphatidyl-glycerol molecules in the K10 ring crystal structure (Murata et al., 2005). In all of these examples detergent micelles were used as membrane mimicking strategies, demonstrating that MS can aid to identify small molecules bound, where high-resolution techniques, such as X-ray crystallography, can have difficulties to resolve them.

With the goal of studying the effect of ligands on MP stability, several recent MS studies have combined high resolution MS, and MD approaches. Marcoux et al. using MS on the ABC transporter P-glycoprotein (P-gp) (Figure 2B), commonly overexpressed in tumor cells, demonstrated that different phosphoglyceride lipids bind more energetically favorable than detergent, which could explain why intact MPs complexed to ligands can be actually detected in the gas phase, prior to protein unfolding. Concomitant binding of ATP and cyclosporine A (CsA) or CL and CsA, triggered greater populations of the smaller conformers which could arise from an unexpected outward-facing form (Marcoux et al., 2013). Laganowsky et al. studied the stability of three membrane transporters using MS. For mechanosensitive channel MscL, four intermediaries' stages were found, and by comparing the apo-state against the lipid bound state, lipid stabilization at each transition could be quantified. Seven different lipids were found and their contributions to MscL stability were similar, except for phosphatidylinositol phosphate (PI) with up to 4 molecules bound, which conferred large increase of stability with each molecule. For water efflux channel AqpZ, similar results were found, with each identified lipid contributing for protein stability with a linear cumulative effect, except for CL, which seems to augment the stability considerably. The ammonia transporter AmtB showed diverse lipid effects on protein stability. While anionic lipids, phosphatidic acid (PA) and phosphatidylserine (PS) did not contribute much to protein stability, CL and particularly phosphatidylglycerol (PG) contributed the most. In the case of PG, stabilization augmented linearly with each molecule bound (up to 4 detected) (Figure 2C). By using this method, lipid contribution to protein stability can be quantified and ranked, and lipids can be classified as crucial elements for protein function (Laganowsky et al., 2014). Finally, Gault et al. presented a MS method, aiming to identify multiple ligands bound to MPs. Considering small mass differences between lipids and drugs, the simultaneous identification of both of them was proven difficult. And yet, this high-resolution Orbitrap-based method allowed the identification not only for concomitant binding of drug and lipids, but also determined the acyl chain length that preferentially binds to the protein. For the outer membrane porin OmpF, using an equimolar mixture of DMPG, DPPG, and POPG, showed more preference toward the longer acyl chain POPG (Figure 2C; Gault et al., 2016).

Considering the same MPs shown in Figures 2B,C X-ray crystallography has been able to identify a much larger number of ligands bound. Among them, only ATP at P-glycoprotein was detected using both X-crystallography and MS methods (Figure 2D). Detergent or lipid molecules can be identified incomplete by X-ray crystallography, because the hydrophobic tail is free to interact with many atoms in the TM region, while the hydrophilic head tend to remain in specific places. However, differences from the expression system and purification experimental conditions cannot be dismissed. From crystal or cryo-EM structures, many densities were not appropriately defined because the bound ligand did not necessarily stay in the same conformation across the many protein crystals needed to obtain their atomic coordinates in the complex. Nevertheless, MS applications on native-like MP-ligand complexes allow testing the effect of multi ligands simultaneously, which is rather difficult to detect via crystal or cryo-EM structures. The ligands tested by MS were observed in 375 different Protein Data Bank (PDB) MP structures (Figure 2E), with the majority represented by CL and cholesterol, that are known to be non-bilayer forming lipids modulating the stability of MPs (van den Brink-van der Laan et al., 2004; Osman et al., 2011; Grouleff et al., 2015).

Altogether, by combining MS with X-ray crystallography, overall stoichiometry, location and stability data can be obtained for the simultaneous effect of several ligands in the protein.

## Future perspectives

One essential question for expanding our understanding of the MS of lipid bound to native-like MP complexes is the precise location of the binding site. As ESI-MS can be combined with LC-MS/MS to identify unambiguously the nature and stoichiometry of the lipids bound, several parallel experimental approaches could be coupled to this methodology. Hydrogen/deuterium exchange mass spectrometry (HDX-MS) of MP-lipid complexes may be combined with docking simulations of the lipid into hypothetical binding sites (Hu et al., 2011) located at hydrophobic pockets or intersubunit interfaces. Those residues present in the binding sites would have a lower exchange compared to other equivalent TM residues located elsewhere. Novel simulation approaches in gas phase, combined with structural information such as shape and size via CCS data (Wang et al., 2010), are needed to characterize the dynamical properties of small molecules bound to native-like MPs. For example, Politis et al. developed a hybrid method combining native MS, IM-MS, and bottom-up approaches (HDX, labeling and cross-linking) with molecular modeling to elucidate the structural arrangement of protein assemblies. While it still uses structural information like low resolution atomic models or cryo-EM mapping as the basis for modeling, the refinement is made through the information obtained in the MS approaches (Politis et al., 2014).

Additionally, the application of nanodiscs can provide a more native lipid environment with a specified size or diameter to obtain functional proteins (Lyukmanova et al., 2012). This approach has been used with techniques like NMR (Susac et al., 2014), SPR (Glück et al., 2011), and MALDI (Marty et al., 2012), and has recently been used to study native-like MPs in gas phase (Hopper et al., 2013). Furthermore, Zhang et al. presented a method using stable isotope labeling by amino acids in cell culture (SILAC) and nanodiscs to identify potential interactions between a target protein and an entire proteome. In this study, 3 MP (SecYEG, MalFGK_2_, and YidC) were screened against the *E. coli* proteome, isolated the interacting proteins by affinity pull-down and analyzed with tandem mass spectrometry, which allowed the recognition of several protein interactions captured from a whole cell extract (Zhang et al., 2012).

## Author contributions

FM, JC, and NB contributed to the writing, data analysis and edition of the manuscript, as well as approved the final version to be published.

### Conflict of interest statement

The authors declare that the research was conducted in the absence of any commercial or financial relationships that could be construed as a potential conflict of interest.
